# Temporo-spatial expression of adrenomedullin and its receptors in the bovine placenta

**DOI:** 10.1186/1477-7827-11-62

**Published:** 2013-07-13

**Authors:** Ken-Go Hayashi, Misa Hosoe, Ryosuke Sakumoto, Toru Takahashi

**Affiliations:** 1 Animal Physiology Research Unit, Division of Animal Science, National Institute of Agrobiological Sciences, Tsukuba 305-8602, Japan; 2 Animal Development and Differentiation Research Unit, Division of Animal Science, National Institute of Agrobiological Sciences, Tsukuba 305-8602, Japan; 3 Present address: Cooperative Department of Veterinary Medicine, Faculty of Agriculture, Iwate University, Morioka 020-8550, Japan

## Abstract

**Background:**

Adrenomedullin (AM) is a potent vasodilator peptide and is also involved in various physiological activities. In humans and rodents, AM is found in the uteroplacental unit and may be responsible for fetal development and maintenance of placental function. This study investigated 1) the mRNA expression patterns of AM and its receptor components (calcitonin receptor-like receptor (CRLR), receptor activity modifying protein (RAMP) 2 and RAMP3) during pregnancy and 2) mRNA and protein localization of AM, CRLR and RAMPs in the bovine placentome.

**Methods:**

For real-time quantitative RT-PCR, bovine uteroplacental tissues were collected from Day 25, 60, 100, 150, 200 and 250 of gestation and separated into uterine caruncle (CAR), intercaruncular endometrium (ICAR), extra-embryonic membranes on Day 25 and cotyledonary villous after Day 60 (EEM-COT) and intercotyledonary chorion (ICOT). *In situ* hybridization and immunohistochemistry was performed to investigate the cellular localization of mRNA and protein of AM, CRLR, RAMP2 and RAMP3 in the placentome on Day 56, 150 and 230 of gestation and interplacentomal tissues on Day 56 of gestation.

**Results:**

*AM* mRNA was highly expressed on Day 200 in EEM-COT, CAR and ICAR. *CRLR* mRNA was highly expressed on Day 60 in all portions. *RAMP2* mRNA was also highly expressed on Day 60 in ICOT and ICAR. In EEM-COT, mRNA expression of *CRLR* and *RAMP2* decreased from Day 150 to 250. *RAMP3* mRNA was highly expressed on Day 150 in EEM-COT, ICOT and ICAR. A distinct AM mRNA and protein signal were only found in trophoblast binucleate cells (BNCs), whereas those of CRLR, RAMP2 and RAMP3 were detected in cotyledonary villous and caruncular epithelial cells. In interplacentomal tissues, AM was detected in BNCs of fetal membrane and a small part of luminal epithelium, endothelial lineage of blood vessels and glandular epithelium of the endometrium. Distinct signals of CRLR, RAMP2 and RAMP3 were found in trophoblast cells, luminal epithelium, stroma under the epithelium, endothelial lineage of blood vessels and glandular epithelium.

**Conclusions:**

Our results indicate that the AM system in the bovine uteroplacental unit may be activated at placentation and transition from the mid to late gestation period. Locally produced AM in the BNCs may play a crucial role in regulation of placental vascular and cellular functions during pregnancy.

## Background

Ruminants have an epitheliochorial-cotyledonary placenta, which is formed at the interface between endometrial caruncles (CAR) and fetal cotyledon (COT) and especially characterizes the migration of trophoblast binucleate cells (BNCs) [1]. Both fetal and maternal components of bovine placenta develop vasculature at the first trimester of gestation, which become more elaborate structures during the course of pregnancy [2]. A variety of angiogenic and vasoactive substances released from vascular endothelial cells and placental cells is thought to regulate local vascular functions in the bovine placenta [3-6].

Adrenomedullin (AM) is a potent vasodilator peptide that was originally isolated from human phaeochromocytoma [7]. Bovine AM consists of 52 amino acids and its sequence is highly conserved with human, porcine, canine, rat and mouse AM [8]. Adrenomedullin acts through a complex of calcitonin receptor-like receptor (CRLR) and its associated receptor activity modifying proteins (RAMPs) [9]. CRLR combines with RAMP2 and RAMP3 to form AM1 receptor and AM2 receptor, respectively. In addition to potent vasodilatory effects, AM is involved in multiple physiological activities such as angiogenesis, apoptosis, inflammation, cell proliferation and endocrine secretion [10-12]. Previous studies have demonstrated that AM function is required for normal fetal growth and placental development in humans and rodents. In the human placenta, AM and CRLR are mainly expressed in syncytiotrophoblast cells and fetal membranes including amnion and extravillous cytotrophoblast cells [13-17]. In the mouse placenta, trophoblast giant cells abundantly express *AM* mRNA [18]. Both *AM* and *RAMP3* mRNA levels in rat placenta were higher in mid than in late gestation [19]. In rats, AM antagonist treatment during early or late gestation decreased placental size, restricted fetal growth and induced deficient placental vascular formation [20,21]. AM heterozygous knockout mice had reduced fertility caused by restricted fetal growth due to a high incidence of abnormal trophoblast cell invasion followed by morphological placental defects [22].

The ruminant placenta has different structures from humans and rodents, which is characterized by non-invasive trophoblast cells that never migrate into the basement membrane of the uterine endometrial unlike the placenta of humans and rodents. Since AM plays a role in various physiological functions such as angiogenesis, tissue remodeling, hormone secretion and placental development, we expect that AM is locally produced and its receptors are also distributed in the bovine placenta. However, there is no detailed information available about the involvement of AM in feto-placental development in ruminants. The objective of this study was 1) to determine mRNA expression patterns of *AM*, *CRLR*, *RAMP2* and *RAMP3* in the bovine uteroplacental unit during pregnancy; and 2) to investigate mRNA and protein localization of AM, CRLR and RAMPs in the bovine placentome and interplacentomal tissues.

## Methods

### Animals and sample collection

Bovine uteroplacental tissues for quantitative real-time PCR (QPCR) were obtained from pregnant Japanese-Black cows in the institute ranch. We collected tissue samples from endometrial tissues of intercaruncular areas (ICAR), extravillous trophoblast of intercotyledonary areas (ICOT) on Day 25 to 28 (Day 25; n = 4 animals), 56 to 65 (Day 60; n = 6 animals), 100 (Day 100; n = 3 animals), 143 to 150 (Day 150; n = 4 animals), 201 to 203 (Day 200; n = 4 animals) and 245 to 259 (Day 250; n = 6 animals) and placentome on Day 60, 100, 150, 200 and 250 of gestation. The placentome were manually separated into two portions, CAR and COT. The day of artificial insemination was designated as Day 1 of gestation. Because it was difficult to isolate COT from the membranes, the extra-embryonic membrane (EEM) on Day 25 contained fetal membrane with a few villi. Collected samples were snap-frozen in liquid nitrogen and stored at -80°C until RNA extraction. Bovine placentome for *in situ* hybridization and immunohistochemistry was collected from Japanese-Black cows immediately after slaughter on Day 56, 150 and 230 of gestation. We also collected the interplacental tissues on Day 56 of gestation. Collected tissues were fixed in 10% formalin (v/v), embedded in paraffin wax, and stored at 4°C until use. All procedures for animal experiments were carried out in accordance with guidelines approved by the Animal Ethics Committee of the National Institute of Agrobiological Sciences for the use of animals (H18-036).

### Quantitative real-time RT-PCR analysis

Total RNA was extracted from each sample using ISOGEN (Nippon Gene, Tokyo, Japan) according to the manufacturer’s instructions. The details of procedures for single-strand cDNA synthesis and QPCR using an Mx3000P QPCR system (Agilent Technologies, Palo Alto, CA, USA) were described previously by our colleague [23]. The primers were designed using the Primer Express computer software program (Applied Biosystems, Foster City, CA, USA) based on the bovine sequences. The primer sequences for each gene are provided in Table 1.

**Table 1:** Details of the primers used for quantitative real-time RT-PCR analysis

**Gene name**	**GenBank accession number**	**Primer**	**Sequences**	**Position**
*AM*	NM_173888	Forward	5′-TCTCAGCGAGATGCAACGTT-3′	1416-1435
		Reverse	5′-CCACAAGAGGCAACTCATCTCT-3′	1492-1471
*CRLR*	NM_001102107	Forward	5′-TCCCAGTTCATCCATCTCTACC-3′	1065-1086
		Reverse	5′-TGCAAATACAGCCACTACAACA-3′	1163-1142
*RAMP2*	NM_001098860	Forward	5′- CAAGAAGGACTGGTGTGATTGG-3′	359-381
		Reverse	5′- AACTCTTCTGCACCCTTTTCCA-3′	443-422
*RAMP3*	NM_001083505	Forward	5′- TGGGTGGCTGCAATGAGAA-3′	113-131
		Reverse	5′- AGAGGTTGCACCACTTCCAGA-3′	217-197
*GAPDH*	U85042	Forward	5′- ACCCAGAAGACTGTGGATGG-3′	444-463
		Reverse	5′-CAACAGACACGTTGGGAGTG-3′	621-602

### In situ hybridization

We investigated mRNA localization of *AM*, *CRLR*, *RAMP2* and *RAMP3* in the bovine placentome on Day 56, 150 and 230 of gestation and in the interplacental tissue on Day 56 of gestation. The details of procedures for preparation of Digoxigenin (DIG)-labeled anti-sense and sense cRNA probes and *in situ* hybridization using an automated Ventana HX System Discovery with a RiboMapKit and a BlueMapKit (Roche Diagnostics, Basel, Switzerland) were described previously by our colleague [24,25]. For *in situ* hybridization, paraffin-embedded samples were sectioned into 7-μm-thick sections. The hybridization signals were detected with a biotin-SP-IgG fraction monoclonal mouse anti-digoxin (200-062-156, Jackson Immuno Research Laboratories, West Grove, PA, USA). The hybridized slides were observed with a Leica DMRE HC microscope (Leica Microsystems, Wetzlar, Germany) and a Nikon Digital Sight DS-Fi1-L2 (Nikon, Tokyo, Japan).

### Immunohistochemistry

Immunohistochemistry for AM, CRLR, RAMP2 and RAMP3 was performed in the bovine placentome on Day 56, 150 and 230 of gestation and in the interplacental tissue on Day 56 of gestation using the automated Ventana HX System Discovery with a DabMapKit (Roche) as described previously in detail by our laboratory [26]. The 7-μm-thick sections were incubated at room temperature with rabbit polyclonal anti-human AM antibody (1.0 mg/ml, T-4131, Peninsula Laboratories, San Carlos, CA, USA), rabbit polyclonal anti-human CRLR antibody (1.0 mg/ml, SP4082P, Acris Antibodies, Herford, Germany), rabbit polyclonal anti-human RAMP2 antibody (0.3 mg/ml, 13223-2-AP, Proteintech Group, Chicago, IL, USA) or rabbit polyclonal anti-human RAMP3 antibody (0.5 mg/ml, LS-B1833, LifeSpan BioSciences, Seattle, WA, USA) diluted 1:10 (anti-CRLR), 1:15 (anti-RAMP3), 1:80 (anti-AM) or 1:100 (anti-RAMP2) in Discovery Ab diluents (Roche) for 2 h. The signals were detected using anti-rabbit IgG-Biotin conjugate (Sigma) diluted 1:500 for 1 h. Negative controls were performed using normal rabbit IgG (5 mg/ml, GTX35035, GeneTex, Irvine, CA, USA) diluted at concentrations equivalent to the primary antibodies. The sections were observed with a Leica DMRE HC microscope (Leica Microsystems) and a Nikon Digital Sight DS-Fi1-L2 (Nikon).

### Statistical analysis

The expression ratio of each gene to *GAPDH* mRNA was calculated to adjust for variations in the QPCR reaction. QPCR results were analyzed using one-way ANOVA followed by the Tukey-Kramer multiple comparison test. Results are presented as the mean ± SEM. Statistical significance is considered to be at *P* < 0.05.

## Results

### mRNA expression of AM, CRLR, RAMP2 and RAMP3 in the bovine uteroplacental unit during pregnancy

**Figure 1:**
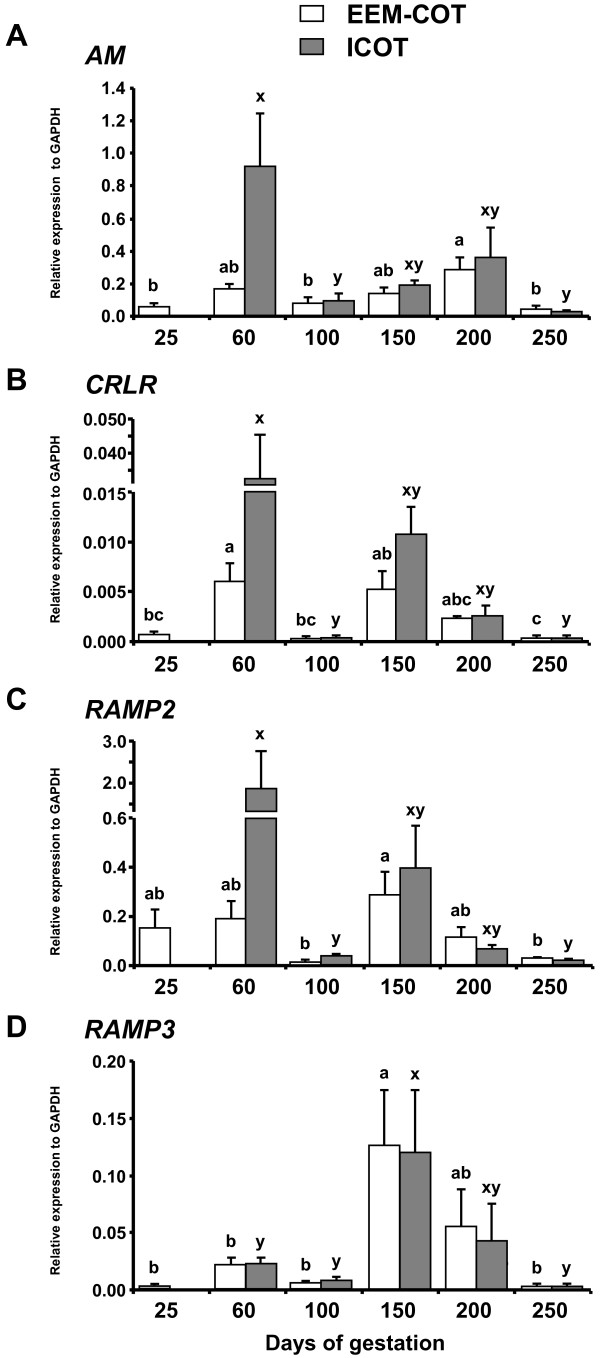
**Expression of *AM*, *CRLR*, *RAMP2 *and *RAMP3 *in EEM-COT and ICOT during bovine pregnancy. **Messenger RNA expression of **(A)***AM*, **(B)***CRLR*, **(C)***RAMP2* and **(D)***RAMP3* in extra-embryonic membrane and cotyledon (EEM-COT) and intercotyledon (ICOT) was analyzed by QPCR. The day of artificial insemination was designated as Day 1 of gestation. The expression of mRNA was normalized to the expression of *GAPDH* mRNA measured in the same RNA preparation. There are no data of *ICOT* mRNA expression on Day 25 of gestation. Data are shown as the mean ± SEM. Values with different letters show significant differences (*P* < 0.05).

Figure 1 shows mRNA expression of *AM*, *CRLR*, *RAMP2* and *RAMP3* in EEM-COT and ICOT during pregnancy. The mRNA expression level of *AM* was higher on Day 200 than on Days 25, 100 and 250 in EEM-COT and higher on Day 60 than on Day 100 and 250 in ICOT (Figure 1A). *CRLR* mRNA expression level in EEM-COT was highest on Day 60 and decreased from Days 150 to 250 with a similar trend in ICOT (Figure 1B). The mRNA expression level of *RAMP2* was higher on Day 150 in EEM-COT and on Day 60 in ICOT than on Day 100 and 250 (Figure 1C). *RAMP3* mRNA expression in EEM-COT and ICOT was higher on Day 150 compared with Days 25, 60, 100 and 250 (Figure 1D).

**Figure 2:**
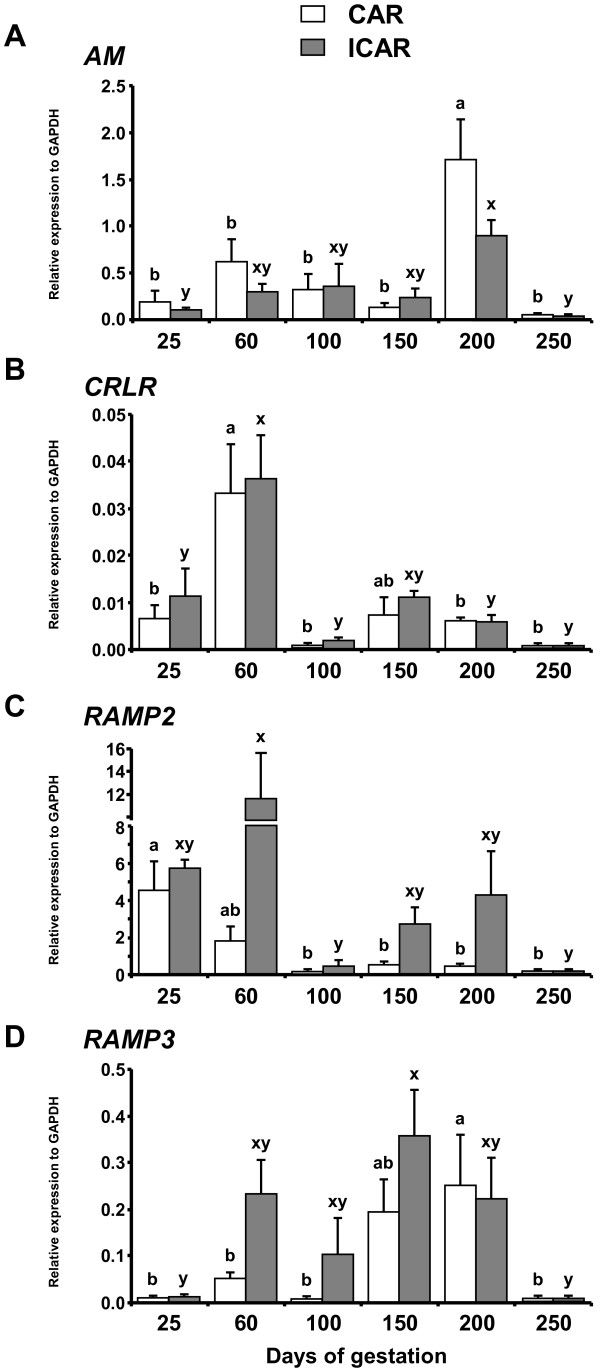
**Expression of *AM*, *CRLR*, *RAMP2 *and *RAMP3 *in CAR and ICAR during bovine pregnancy. **Messenger RNA expression of **(A)***AM*, **(B)***CRLR*, **(C)***RAMP2* and **(D)***RAMP3* in caruncle (CAR) and intercaruncle (ICAR) was analyzed by QPCR. The day of artificial insemination was designated as Day 1 of gestation. The expression of mRNA was normalized to the expression of *GAPDH* mRNA measured in the same RNA preparation. Data are shown as the mean ± SEM. Values with different letters show significant differences (*P* < 0.05).

Figure 2 shows mRNA expression of *AM*, *CRLR*, *RAMP2* and *RAMP3* in CAR and ICAR during pregnancy. *AM* mRNA expression level was higher on Day 200 than on other gestation days and higher on Day 200 than on Days 25 and 250 in CAR and ICAR respectively (Figure 2A). The *CRLR* mRNA expression level in CAR and ICAR were higher on Day 60 than on Day 25, 100, 200 and 250 (Figure 2B). In ICAR, *RAMP2* mRNA expression level was higher on Day 60 than on Day 100 and 250 and in CAR was highest on Day 25 (Figure 2C). *RAMP3* mRNA expression in CAR increased from Day 100 to Day 200 then decreased on Day 250 (Figure 2D). In ICAR, *RAMP3* mRNA expression level was higher on Day 150 than on Day 25 and 250 (Figure 2D). Table 2 shows the summary of mRNA expression profiles of *AM*, *CRLR*, *RAMP2* and *RAMP3* in bovine uteroplacental tissues.

**Table 2:** **Summary of mRNA expression profiles of *AM*, *CRLR*, *RAMP2 *and *RAMP3 *in bovine utero-placental tissues analyzed by QPCR**

**Genes**	**Tissues**	**Days of gestation**					
		**25**	**60**	**100**	**150**	**200**	**250**
*AM*	COT	**+**	**+**	**+**	**+**	**++**	**+**
	ICOT	NA	**+++**	**+**	**+**	**++**	**+**
	CAR	**+**	**++**	**+**	**+**	**+++**	**+**
	ICAR	**+**	**+**	**+**	**+**	**++**	**+**
*CRLR*	COT	**+**	**++**	**+**	**++**	**+**	**+**
	ICOT	NA	**+++**	**+**	**++**	**+**	**+**
	CAR	**+**	**+++**	**+**	**+**	**+**	**+**
	ICAR	**++**	**+++**	**+**	**++**	**+**	**+**
*RAMP2*	COT	**++**	**++**	**+**	**++**	**+**	**+**
	ICOT	NA	**+++**	**+**	**++**	**+**	**+**
	CAR	**++**	**+**	**+**	**+**	**+**	**+**
	ICAR	**++**	+++	+	++	++	+
*RAMP3*	COT	+	+	+	+++	++	+
	ICOT	NA	+	+	+++	++	+
	CAR	+	+	+	+++	+++	+
	ICAR	+	+++	++	+++	+++	+

+++, strong expression; ++, moderate expression; +, slight expression; NA, sample not available.

### mRNA localization of AM, CRLR, RAMP2 and RAMP3 in bovine placentome on Day 56, 150 and 230 of gestation

**Figure 3:**
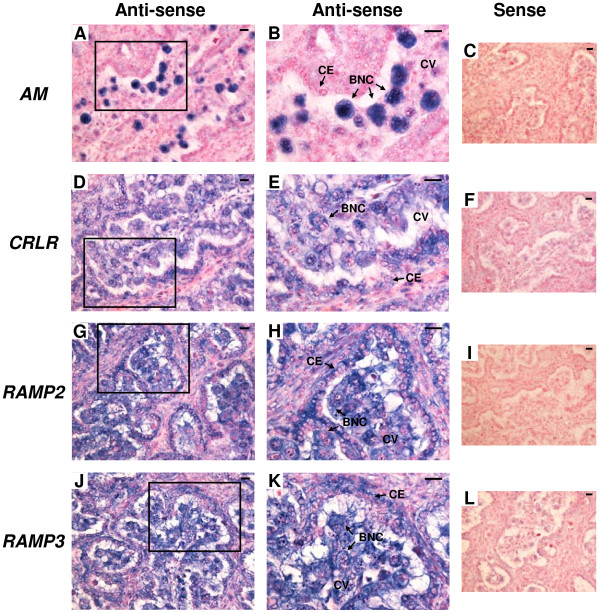
**mRNA localization of *AM*, *CRLR*, *RAMP2 *and *RAMP3 *in the bovine placentome on day 56 of gestation. (A**, **D**, **G** and **J)** Digoxigenin (DIG)-labeled anti-sense cRNA probes were used. **(B**, **E**, **H** and **K)** enlarged images of frames in **A**, **D**, **G** and **J**, respectively. **(C**, **F**, **I** and **L)** DIG-labeled sense cRNA probes were used. Sections (7 μm) of bovine placentome on Day 56 of gestation were hybridized with each probe. *AM* mRNA **(A** and **B)** was detected in only in BNCs. mRNA expression of *CRLR* **(D** and **E)***, RAMP2* **(G** and **H)** and *RAMP3* **(J** and **K)** were detected in cotyledonary villous and CEs. BNC, trophoblast binucleate cell; CE, caruncular epithelial cell; CV, cotyledonary villous. Scale bars = 20 μm.

**Figure 4:**
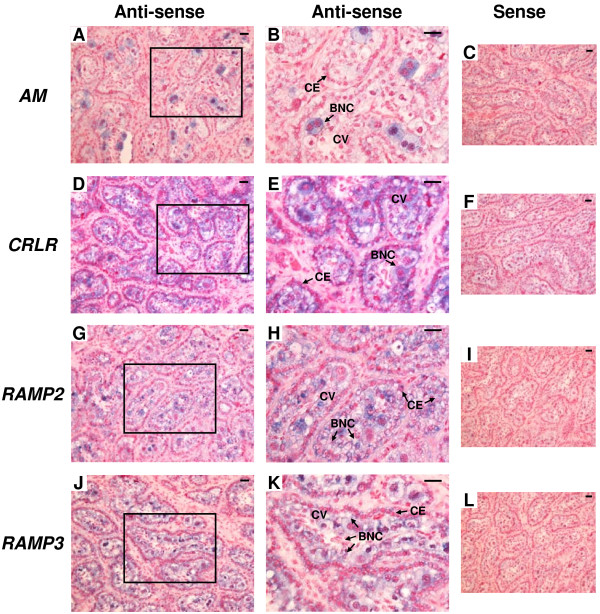
**mRNA localization of *AM*, *CRLR*, *RAMP2 *and *RAMP3 *in the bovine placentome on day 150 of gestation. (A**, **D**, **G** and **J)** Digoxigenin (DIG)-labeled anti-sense cRNA probes were used. **(B**, **E**, **H** and **K)** enlarged images of frames in **A**, **D**, **G** and **J**, respectively. **(C**, **F**, **I** and **L)** DIG-labeled sense cRNA probes were used. Sections (7 μm) of bovine placentome on Day 150 of gestation were hybridized with each probe. *AM* mRNA **(A** and **B)** was detected in only BNCs. mRNA expression of *CRLR* **(D** and **E)**, *RAMP2* **(G** and **H)** and *RAMP3* **(J** and **K)** was detected in cotyledonary villous and CEs. BNC, trophoblast binucleate cell; CE, caruncular epithelial cell; CV, cotyledonary villous. Scale bars = 20 μm.

**Figure 5:**
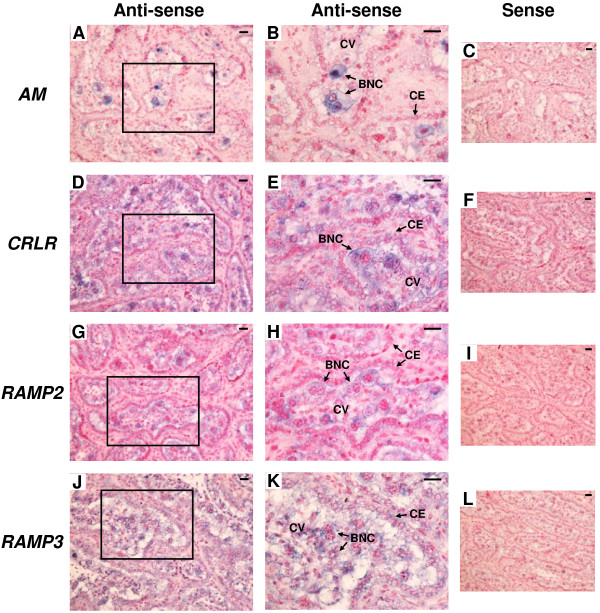
**mRNA localization of *AM*, *CRLR*, *RAMP2 *and *RAMP3 *in the bovine placentome on day 230 of gestation. (A**, **D**, **G** and **J)** Digoxigenin (DIG)-labeled anti-sense cRNA probes were used. **(B**, **E**, **H** and **K)** enlarged images of frames in **(A**, **D**, **G** and **J)** respectively. **(C**, **F**, **I** and **L)** DIG-labeled sense cRNA probes were used. Sections (7 μm) of bovine placentome on Day 230 of gestation were hybridized with each probe. *AM* mRNA **(A** and **B)** was detected in only BNCs. mRNA expression of *CRLR* **(D** and **E)**, *RAMP2* **(G** and **H)** and *RAMP3* **(J** and **K)** was detected in cotyledonary villous and CEs. BNC, trophoblast binucleate cell; CE, caruncular epithelial cell; CV, cotyledonary villous. Scale bars = 20 μm.

The results of *in situ* hybridization for *AM*, *CRLR*, *RAMP2* and *RAMP3* in the bovine placentome on Day 56, 150 and 230 of gestation are shown in Figures 3, 4 and 5, respectively. Throughout gestation, *AM* mRNA signal was detected only in BNCs (Figure 3A and B, Figure 4A and B and Figure 5A and B). *CRLR* mRNA was detected in both cotyledonary villous including BNCs and caruncular epithelial cells (Figure 3D and E, Figure 4D and E and Figure 5D and E). Both *RAMP2* and *RAMP3* mRNA were also found in both the cotyledonary villi, including BNCs and the caruncular epithelial cells (Figure 3G,H,J and K, Figure 4G,H,J and K and Figure 5G,H,J and K). No significant signal was detected with any of the sense probes (Figure 3C,F,I and L, Figure 4C,F,I and L and Figure 5C,F,I and L).

### mRNA localization of AM, CRLR, RAMP2 and RAMP3 in bovine interplacentomal tissues on Day 56 of gestation

**Figure 6:**
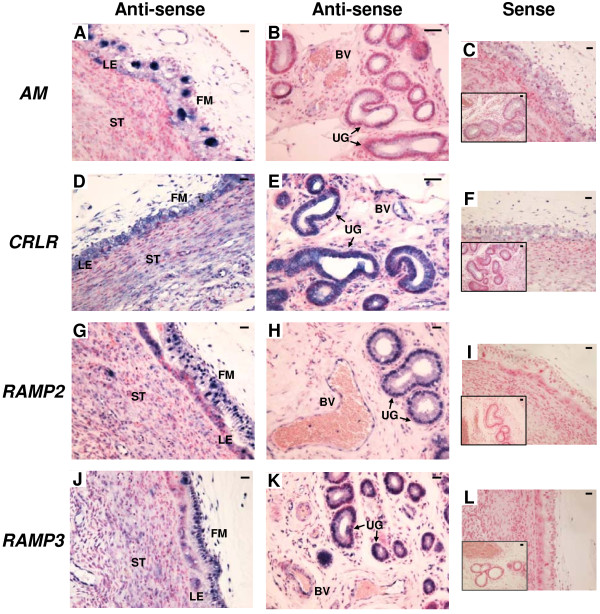
**mRNA localization of *AM*, *CRLR*, *RAMP2 *and *RAMP3 *in the bovine interplacentomal tissues on day 56 of gestation. (A**, **B**, **D**, **E**, **G**, **H**, **J** and **K)** Digoxigenin (DIG)-labeled anti-sense cRNA probes were used. **(C**, **F**, **I** and **L)** DIG-labeled sense cRNA probes were used. Sections (7 μm) of bovine interplacentomal tissues on Day 56 of gestation were hybridized with each probe. **(A**, **D**, **G** and **J)** fetal membrane and endometrium. **(B**, **E**, **H** and **K)** blood vessels and uterine glands of the endometrium. The small panels within **C**, **F**, **I** and **L** show the portion of the blood vessels and uterine glands. FM, fetal membrane; LE, endometrial luminal epithelium; ST, stroma; BV, blood vessel; UG, uterine gland. Scale bars = 20 μm.

Figure 6 shows the results of *in situ* hybridization for *AM*, *CRLR*, *RAMP2* and *RAMP3* in the bovine interplacentomal tissues on Day 56 of gestation. *AM* mRNA was detected in BNCs of fetal membrane and a part of luminal epithelium (Figure 6A). It was also found in a small part of endothelial lineage of blood vessels and glandular epithelium of the endometrium (Figure 6B). *CRLR*, *RAMP2* and *RAMP3* mRNA were found in trophoblast cells including the BNCs, luminal epithelium, stroma under the epithelium, endothelial lineage of blood vessels and glandular epithelium (Figure 6D,E,G,H,J and K). No significant signal was detected with any of the sense probes (Figure 6C,F,I and L).

### Protein localization of AM, CRLR, RAMP2 and RAMP3 in the bovine placentome on Day 56, 150 and 230 of gestation

**Figure 7:**
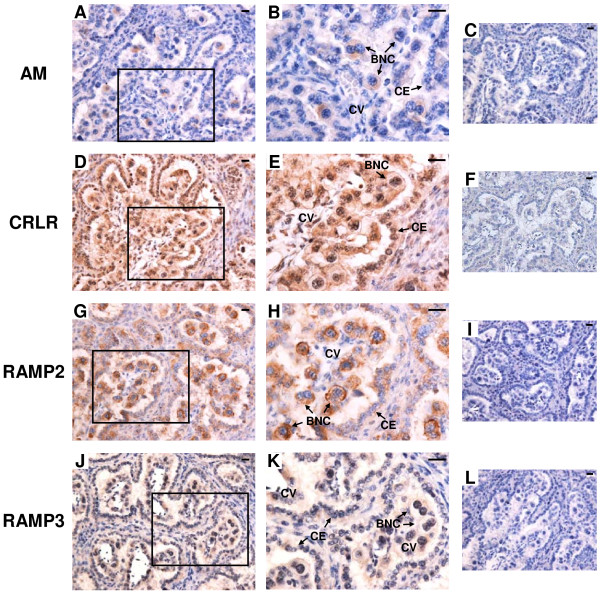
**Protein localization of AM, CRLR, RAMP2 and RAMP3 in the bovine placentome on day 56 of gestation. **Protein localization of **(A)** AM, **(D)** CRLR, **(G)** RAMP2 and **(J)** RAMP3 was detected by immunohistochemistry. **(B**, **E**, **H** and **K)** enlarged images of frames in **A**, **D**, **G** and **J**, respectively. Sections (7 μm) of the bovine placentome on Day 56 of gestation were incubated with anti-human AM, anti-human CRLR, anti-human RAMP2 and anti-human RAMP3 polyclonal antibodies. A distinct AM signal was found only in BNCs. Protein localization of CRLR, RAMP2 and RAMP3 was detected in cotyledonary villous and CEs. **(C**, **F**, **I** and **L)** no signal was detected in the negative control sections using normal rabbit IgG. BNC, trophoblast binucleate cell; CE, caruncular epithelial cell; CV, cotyledonary villous. Scale bars = 20 μm.

**Figure 8:**
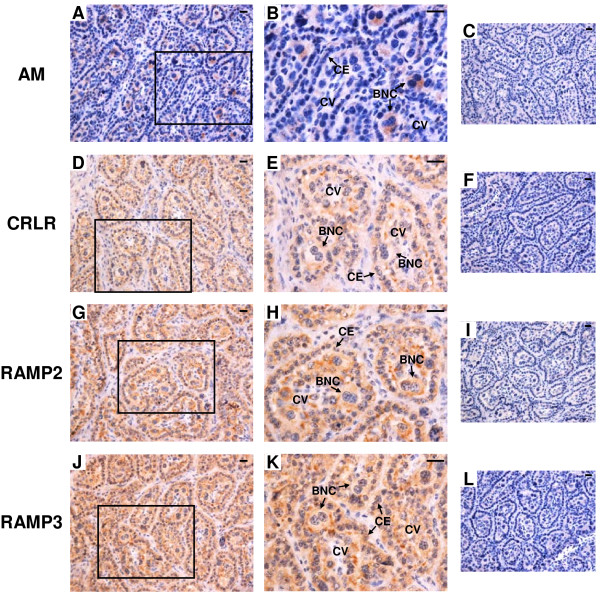
**Protein localization of AM, CRLR, RAMP2 and RAMP3 in the bovine placentome on day 150 of gestation. **Protein localization of **(A)** AM, **(D)** CRLR, **(G)** RAMP2 and **(J)** RAMP3 was detected by immunohistochemistry. **(B**, **E**, **H** and **K)** enlarged images of frames in **A**, **D**, **G** and **J**, respectively. Sections (7 μm) of the bovine placentome on Day 150 of gestation were incubated with anti-human AM, anti-human CRLR, anti-human RAMP2 and anti-human RAMP3 polyclonal antibodies. A positive AM signal was found in BNCs. Protein localization of CRLR, RAMP2 and RAMP3 was detected in cotyledonary villous and CEs. **(C**, **F**, **I** and **L)** no signal was detected in the negative control sections using normal rabbit IgG. BNC, trophoblast binucleate cell; CE, caruncular epithelial cell; CV, cotyledonary villous. Scale bars = 20 μm.

**Figure 9:**
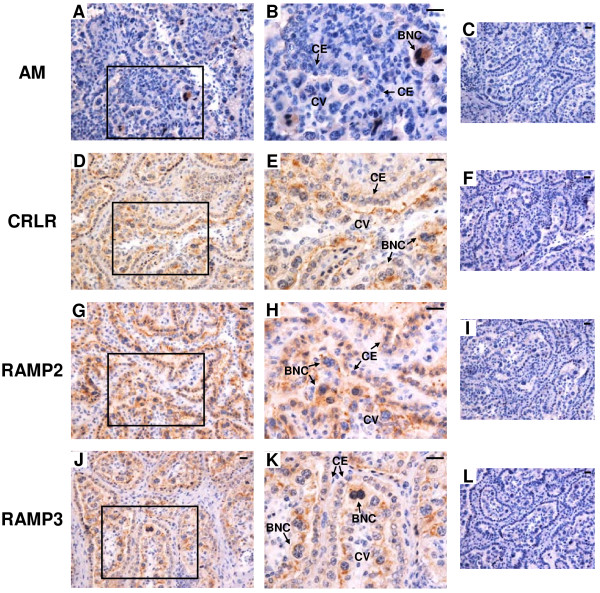
**Protein localization of AM, CRLR, RAMP2 and RAMP3 in the bovine placentome on day 230 of gestation. **Protein localization of **(A)** AM, **(D)** CRLR, **(G)** RAMP2 and **(J)** RAMP3 was detected by immunohistochemistry. **(B**, **E**, **H** and **K)** enlarged images of frames in **A**, **D**, **G** and **J**, respectively. Sections (7 μm) of the bovine placentome on Day 230 of gestation were incubated with anti-human AM, anti-human CRLR, anti-human RAMP2 and anti-human RAMP3 polyclonal antibodies. Protein localization of AM was found in BNCs. CRLR, RAMP2 and RAMP3 was detected in cotyledonary villous and CEs. **(C**, **F**, **I** and **L**) no signal was detected in the negative control sections using normal rabbit IgG. BNC, trophoblast binucleate cell; CE, caruncular epithelial cell; CV, cotyledonary villous. Scale bars = 20 μm.

The results of immunohistochemistry for *AM*, *CRLR*, *RAMP2* and *RAMP3* in the bovine placentome on Day 56, 150 and 230 of gestation are shown in Figures 7, 8 and 9, respectively. Throughout gestation, a distinct AM signal was found only in BNCs (Figure 7A and B, Figure 8A and B and Figure 9A and B). CRLR, RAMP2 and RAMP3 proteins were detected in cotyledonary villous, including BNCs, and caruncular epithelial cells (Figure 7D,E,G,H,J and K, Figure 8D,E,G,H,J and K and Figure 9D,E,G,H,J and K).

### Protein localization of AM, CRLR, RAMP2 and RAMP3 in the bovine interplacentomal tissues on Day 56 of gestation

**Figure 10:**
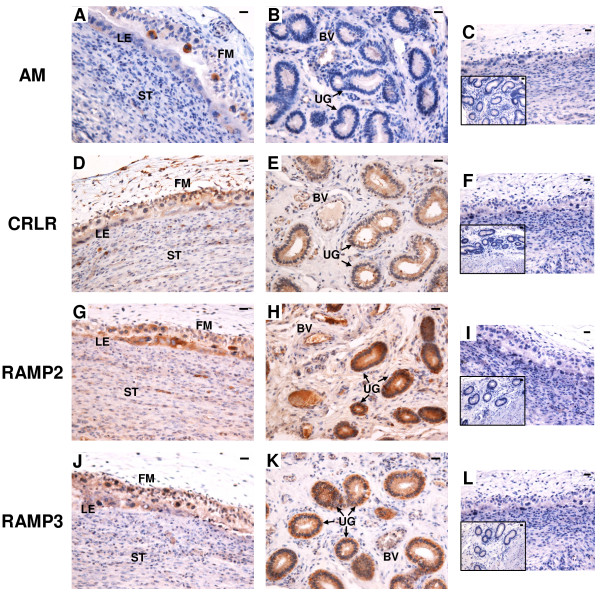
**Protein localization of AM, CRLR, RAMP2 and RAMP3 in the bovine interplacentomal tissues on day 56 of gestation.** Protein localization of **(A** and **B)** AM, **(D** and **E)** CRLR, **(G** and **H)** RAMP2 and **(J** and **K)** RAMP3 was detected by immunohistochemistry. **(A**, **D**, **G** and **J)** fetal membrane and endometrium. **(B**, **E**, **H** and **K)** blood vessels and uterine glands of the endometrium. Sections (7 μm) of the bovine interplacentomal tissues on Day 56 of gestation were incubated with anti-human AM, anti-human CRLR, anti-human RAMP2 and anti-human RAMP3 polyclonal antibodies. **(C**, **F**, **I** and **L)** no signal was detected in the negative control sections using normal rabbit IgG. The small panels within C, F, I and L show the portion of the blood vessels and uterine glands. FM, fetal membrane; LE, endometrial luminal epithelium; ST, stroma; BV, blood vessel; UG, uterine gland. Scale bars = 20 μm.

Figure 10 shows the results of *in situ* hybridization for *AM*, *CRLR*, *RAMP2* and *RAMP3* in the bovine interplacentomal tissues on Day 56 of gestation. Immunoreactive AM protein was detected in BNCs of fetal membrane and a part of luminal epithelium (Figure 6A). A weak AM staining was found in endothelial lineage of blood vessels and glandular epithelium of the endometrium (Figure 10B). CRLR, RAMP2 and RAMP3 positive staining were found in trophoblast cells, luminal epithelia, stroma under the epithelium, endothelial lineage of blood vessels and glandular epithelium (Figure 10D,E,G,H,J and K).

## Discussion

This is the first study to show that AM and its receptors are expressed in the bovine uteroplacental unit and that their mRNA expression pattern changes considerably during the course of pregnancy. We also demonstrated for the first time that AM mRNA and protein in the bovine placentome were detected only in BNCs, whereas those of CRLR, RAMP2 and RAMP3 were localized in trophoblast cells, including the BNCs, and caruncular epithelial cells. In interplacentomal tissues, mRNA and protein of AM were mainly detected in BNCs of fetal membrane and CRLR, RAMP2 and RAMP3 were found in fetal membrane, luminal epithelium, stroma under the epithelium, endothelial lineage of blood vessels and glandular epithelium. The results of *in situ* hybridization and immunohistochemistry were almost match, suggesting transcripted and translated AM, CRLR and RAMP2 proteins continue to localize in the same cells in bovine utero-placenta throughout gestation period. It seems that locally produced AM is an important factor in the regulation of bovine placental function, as has been previously suggested in human and mouse studies [10].

In this study, the mRNA expression profiles of *AM*, *CRLR*, *RAMP2* and *RAMP3* in the uteroplacental unit were summarized as follows: *AM* mRNA was expressed in all tissues from the beginning of placentation to mid gestation transition (Days 60–200). *CRLR* mRNA was lowest expression but most abundant in maternal tissue at the beginning of placentation. *RAMP2* mRNA was also most abundant in maternal tissue at the beginning of placentation whereas *RAMP3* mRNA was present in the fetal and maternal tissues more abundantly mid to late gestation. *AM* mRNA in ICOT and *CRLR* and *RAMP2* mRNA in all regions were highly expressed on Day 60 of gestation. AM promotes the migration and invasion of endothelial cells through CRLR/RAMP2 and CRLR/RAMP3 receptors [27]. Homozygous knockout of *RAMP2* causes embryonic death in the mouse due to severe vascular abnormality, suggesting that the CRLR/RAMP2 receptor is required for angiogenesis [28]. Both maternal and fetal vasculature of the bovine placentome at the third and fourth month of gestation are still immature and consist only of simple capillary loops [2]. Although changes in mRNA expression of two of the primary angiogenic factors, fibroblast growth factor (FGF) and vascular endothelial growth factor (VEGF), have been reported in the bovine placentome during pregnancy, VEGF and its receptors on both the fetal and maternal side of the placentome increase after Day 80 of gestation and FGF1 and FGF type 2 receptor in the total placentome increase from around Day 80 until Day 200 of gestation [5]. Therefore, increased *CRLR* and *RAMP2* mRNA expression in the bovine uteroplacental unit at Day 60 of gestation may be involved in the stimulation of angiogenesis at an early stage of placentation before the increase in VEGF and FGF.

*AM* and *RAMP3* mRNA were highly expressed in EEM-COT, CAR and ICAR from Day 150 to Day 200 as compared with early or mid-gestation. The AM signal during this period is likely to act via the CRLR/RAMP3 receptor. The weight and length of bovine placentomes increase exponentially and their growth rates peak at Day 200 of gestation [29]. In addition, bovine uterine artery blood flow increases linearly throughout gestation, suggesting an increase in blood supply to the placenta [30]. In the bovine uteroplacental unit, gene expression of several major vasoactive substances such as endothelin-1 system, angiotensin II system and endothelial nitric oxide (NO) synthase increase during the mid-gestation period [3,4,6]. The AM-induced vasodilatation is partially mediated through NO release [31]. Locally produced AM in bovine placenta may regulate the vascular tonus in response to increasing blood supply by coordinating with other vasoactive substances to support placental development and feto-maternal exchange. Treatment with AM antagonist during late gestation in the rat causes necrosis in the decidua and labyrinthine trophoblast resulting in decreased placental and fetal weight and an increased incidence of fetal reabsorption [20]. One possible reason for these abnormalities is deficient vascular development in the placenta [20]. Therefore, it is plausible that AM plays certain roles in not only functional, but also morphological regulation of uteroplacental vasculature during mid to late gestation in bovine.

We revealed by *in situ* hybridization and immunohistochemistry that the BNC is the primary source of AM production in the bovine placenta. In addition, CRLR and RAMPs are localized in same cell types within bovine uteroplacental tissues. This suggests that CRLR and RAMPs form receptor complex and exist as functional AM receptors in these cells. The secreted AM may act on fetal and maternal placental cells through an autocrine/paracrine mechanism. This finding is in agreement with previous report that *AM* mRNA is most highly expressed in trophoblast giant cells in the mouse placenta [18]. The bovine BNC secretes various placental-specific molecules such as placental lactogen (PL), pregnancy-associated glycoproteins (PAGs) and prolactin-related proteins (PRPs) in COT and ICOT, which play a crucial role in the regulation of placentation, maintenance of pregnancy and stimulation of fetal growth [32]. Some of these molecules show a similar temporal expression profile with AM during pregnancy, that is the mRNA expression of *PRP1* in ICOT peaks at Day 60 of gestation and *PL*, *PAG1*, *PAG9* and *PRP-VII* in COT begins to increase at mid gestation [24,33]. Thus, we speculate that AM may be involved in the regulation of secretory function of the BNC to interact with these placental-specific molecules in both placentome and interplacentomal regions. In addition, it has been reported that AM affects the secretory activities of endocrine organs including the pituitary gland, adrenal cortex and ovary [34-38]. In rats, AM inhibited FSH-induced estradiol secretion in follicles and also suppressed eCG-stimulated progesterone release in corpus luteum [37]. *In vitro* treatment of preantral follicular culture with AM increased estradiol production [38]. The regulation of progesterone production by AM in corpus luteum in culture was pregnancy-stage dependent, inhibitory at early and late pregnancy but stimulatory at mid pregnancy [38]. Since the utero-placenta is a major source of estrogen secretion during bovine pregnancy [39,40], AM may act as an important regulator in steroids production to maintain pregnancy.

Adrenomedullin enhances both cell invasion and proliferation via CRLR and RAMP2 in human choriocarcinoma cells [41]. In mice, invasive trophoblast giant cells show dramatic upregulation of AM genes compared with undifferentiated trophectoderm cells, indicating the involvement of AM in trophoblast invasion and guidance of developing placental tissue [22]. The bovine BNC is known to appear around Day 20 of gestation and migrate and fuse with endometrial epithelium from the trophoblast epithelium throughout gestation [1]. *AM*, *CRLR* and *RAMP*2 mRNA were expressed in BNCs throughout gestation, suggesting that AM may also have a regulatory effect on migration, proliferation and/or turnover of the BNC itself during bovine placental development.

Treatment with AM antagonist during early rat pregnancy causes severely retarded placental development and restricted fetal growth through apoptosis of the placenta and uterus [21]. This activation of apoptosis is due to decreased antiapoptotic protein Bcl-2 levels and increased mitochondrial proapoptotic Bcl2-associated X protein (Bax) levels with a decrease in cytochrome C levels in both the placenta and uterus [21]. In the bovine placenta, mRNA expression of Bcl-2 related antiapoptotic protein *Bcl2A1* in bovine COT is higher on Day 60 and 150 of gestation than in EEM on Day 28 of gestation and the expression ratio between Bcl2A1 and BAX is highest on Day 60 of gestation [25]. In addition, the mean number of apoptotic cells in bovine fetal and maternal placenta increases significantly from the first to the third trimester [42]. Both *CRLR* and *RAMP2* mRNA in the bovine placenta may also be involved in regulation of local apoptosis for adequate placentation.

In the present study, AM, CRLR, RAMP2 and RAMP3 mRNA and protein were also found in interplacentomal endometrium (ICAR) by both QPCR and histological studies. It has been reported that uterine secretions are essential for survival and development of the embryo and associated extraembryonic membranes [43] and uterine artery blood flow increase throughout bovine pregnancy [30]. The AM system may affect the regulation of cellular remodeling of luminal epithelium, angiogenesis, vascular permeability and uterine gland function via AM receptors within endometrium. Further studies are required to determine the functional role of AM system in bovine placental development and maintenance of pregnancy.

## Conclusions

In conclusion, our results indicate that the AM system in the bovine uteroplacental unit may be activated at the beginning of placentation and transition from the mid to late gestation period. Locally produced AM in the BNC may play a crucial role in the regulation of placental vascular and cellular functions by autocrine/paracrine action during pregnancy.

## Competing interests

The authors declare that they have no competing interests.

## Authors’ contributions

KGH participated in the design of the study, collected the materials, carried out all experiments and drafted the manuscript. MH was responsible for all animal care, collected the materials and helped to carry out all experiments. RS collected the materials and helped to carry out QPCR and *in situ* hybridization. TT supervised the study, collected the materials and helped to draft the manuscript. All authors read and approved the final manuscript.
